# Identification and Structural Characterization of Novel Chondroitin/Dermatan Sulfate Hexassacharide Domains in Human Decorin by Ion Mobility Tandem Mass Spectrometry

**DOI:** 10.3390/molecules27186026

**Published:** 2022-09-15

**Authors:** Mirela Sarbu, Raluca Ica, Edie Sharon, David E. Clemmer, Alina D. Zamfir

**Affiliations:** 1 Department of Condensed Matter, National Institute for Research and Development in Electrochemistry and Condensed Matter, 300569 Timisoara, Romania; 2 Department of Physics, West University of Timisoara, 300223 Timisoara, Romania; 3 Department of Chemistry, The College of Arts & Science, Indiana University, Bloomington, IN 47405-7102, USA; 4 Department of Technical and Natural Sciences, “Aurel Vlaicu” University of Arad, 310330 Arad, Romania

**Keywords:** chondroition/dermatan sulfate, human decorin, ion mobility mass spectrometry, tandem mass spectrometry, structural analysis, sulfation code

## Abstract

Chondroitin sulfate (CS) and dermatan sulfate (DS) are found in nature linked to proteoglycans, most often as hybrid CS/DS chains. In the extracellular matrix, where they are highly expressed, CS/DS are involved in fundamental processes and various pathologies. The structural diversity of CS/DS domains gave rise to efforts for the development of efficient analytical methods, among which is mass spectrometry (MS), one of the most resourceful techniques for the identification of novel species and their structure elucidation. In this context, we report here on the introduction of a fast, sensitive, and reliable approach based on ion mobility separation (IMS) MS and MS/MS by collision-induced dissociation (CID), for the profiling and structural analysis of CS/DS hexasaccharide domains in human embryonic kidney HEK293 cells decorin (DCN), obtained after CS/DS chain releasing by β-elimination, depolymerization using chondroitin AC I lyase, and fractionation by size-exclusion chromatography. By IMS MS, we were able to find novel CS/DS species, i.e., under- and oversulfated hexasaccharide domains in the released CS/DS chain. In the last stage of analysis, the optimized IMS CID MS/MS provided a series of diagnostic fragment ions crucial for the characterization of the misregulations, which occurred in the sulfation code of the trisulfated-4,5-Δ-GlcAGalNAc[IdoAGalNAc]_2_ sequence, due to the unusual sulfation sites.

## 1. Introduction

Proteoglycans (PGs) represent heavily glycosylated proteins, expressed predominantly in the extracellular matrix (ECM) where they are implicated in key biological processes [1,2]. PGs consist of a core protein covalently linked to one or more linear, heterogeneous, and sulfated polysaccharide chains of *O*-glycan type, known as glycosaminoglycans (GAGs). GAG chain(s) account for more than a half of the PG molecular weight, therefore GAGs trigger most of the biological events in which PGs are involved, mediate the interactions with other proteins, such as the growth factors, and determine the general properties of the respective proteoglycan [3,4,5].

Among GAGs, chondroitin sulfate (CS) and dermatan sulfate (DS), also known as galactosaminoglycans, are found linked to a variety of PGs, most often as hybrid CS/DS chains, in which CS motifs are interspersed with DS sequences. At the ECM level, CS and DS are responsible for fundamental cell- and tissue-related events and various pathologies, including malignant transformations [1,6,7,8,9,10].

Structurally, CS consists of GlcA β1-3 GalNAc disaccharide repeats, where GalNAc represents *N*-acetyl-galactosamine and GlcA is D-glucuronate. GlcA β1-3 GalNAc disaccharides are sulfated and linked by β1-4 glycosidic bond; DS disaccharide repeating units have a similar composition, with GlcA epimerized to IdoA, which is L-iduronate. In regularly sulfated domains, which, usually, are the most numerous in a chain, the sulfate ester group is located at the GalNAc moiety. In such cases, the number of the sulfates equals the number of the disaccharides existing in the composition of the chain. Nevertheless, irregularly sulfated motifs, having the sulfates located at the hexuronic acid and/or GalNAc in a high variety of arrangements that are energetically and structurally favorable, were reported in the last years [11,12,13]. Given that in CS/DS domains, sulfation is generated by a non-template driven process, the number of sulfates may vary from a sequence to another, thus, undersulfated and oversulfated domains may also occur and play major roles, including the mediation of the interactions with specific proteins, an event for which the sulfation status is a crucial determinant [9,12]. Hence, the biological functions of CS/DS-PGs are strongly influenced by the sulfation code, i.e., the number of sulfate groups and their sites. Since a large proportion of brain ECM is composed of PGs, CS/DS sulfation is also correlated with the functional diversity of neurons, brain development, and repair, as well as severe brain conditions [14,15,16,17].

The elevated structural diversity of the CS/DS sequences, the variability of sulfation even within a single GAG chain, in which the sulfate group may occur not only at GalNAc but also at IdoA/GlcA, as well as the biomedical importance of these species, stimulated the development of an array of analytical methods for detection and detailed structure elucidation of CS/DS motifs in various PGs.

Mass spectrometry (MS) with either nanoelectrospray ionization (nanoESI) or microfluidics-based ESI was shown to be one of the most efficient techniques able to provide *de novo* structural information on oligosaccharides resulting after the depolymerization of the GAG chain, as well as some of their interactions with specific proteins [18,19,20,21,22]. However, several limitations of the MS were signaled as well; they derive mainly from the necessity to depolymerize the long CS/DS chain and analyze the mixture of shorter chains differing in their length and sulfation code. The MS challenges [23,24] are related to the difficulties to achieve: (i) a fair ionization yield of species in such mixtures; (ii) the detection of single CS/DS motif in a multicomponent sample encompassing chains of different sulfation pattern; and (iii) the discrimination of isobaric structures. Thereby, the investigation of various aspects related to CS, DS, or hybrid CS/DS, required the combination of MS with efficient separation techniques in the category of high-performance capillary electrophoretic (CE) or liquid chromatographic (LC) methods [25,26,27,28,29]. The most modern platforms involved liquid-based separation techniques coupled to tandem MS (MS/MS) and automated peak detection by dedicated software [25]. Although such integrated systems represented an advanced technology in the MS-based glycosaminoglycomics, and improved the consistency of the data on the composition of natural GAGs, a number of shortcomings, such as the incompatibility of solvents, flow rates, voltages, and the designing of the coupling interfaces, along with the persistent difficulty in discriminating some isobaric GAG species, still persisted. On the other hand, recently, ion mobility separation (IMS) mass spectrometry has emerged as one of the most resourceful MS techniques in targeted and untargeted omics-related workflows since, based on the properties of the transport driven by the electric field, the method is able to separate isomers, isobars and conformers according to their mobility, and provide detailed information on the stoichiometry, topology and structure as no other MS-based system. Earlier work on the implementation of IMS MS in glycopeptide and glycolipid analysis [30,31,32,33,34,35], as well as the recent reports on the IMS MS and MS/MS of heparin/heparan sulfate and their interactions [36,37,38] have indicated that, in conjunction with effective fragmentation techniques, IMS MS results in a powerful and highly sensitive method capable of separation, detection by MS and structural characterization by MS/MS of even minor compounds in a complex mixture of glycans in a single run and on a single instrument.

In this context, we report here on the introduction of a rapid, highly sensitive, and reliable approach in a single run, based on IMS MS and MS/MS by collision-induced dissociation (CID), for screening and structural analysis of CS/DS derived from human decorin (DCN). By IMS MS and CID MS/MS, we were able to characterize in details CS/DS hexasaccharides in human embryonic kidney HEK293 cells DCN obtained after CS/DS chain depolymerization by chondroitin AC I lyase and to detect sequences that were never found before in the hexasaccharide domains of the investigated decorin.

## 2. Results and Discussion

### 2.1. IMS MS Analysis of Hexasaccharide Domains in CS/DS from HEK293 DCN

**Figure 1:**
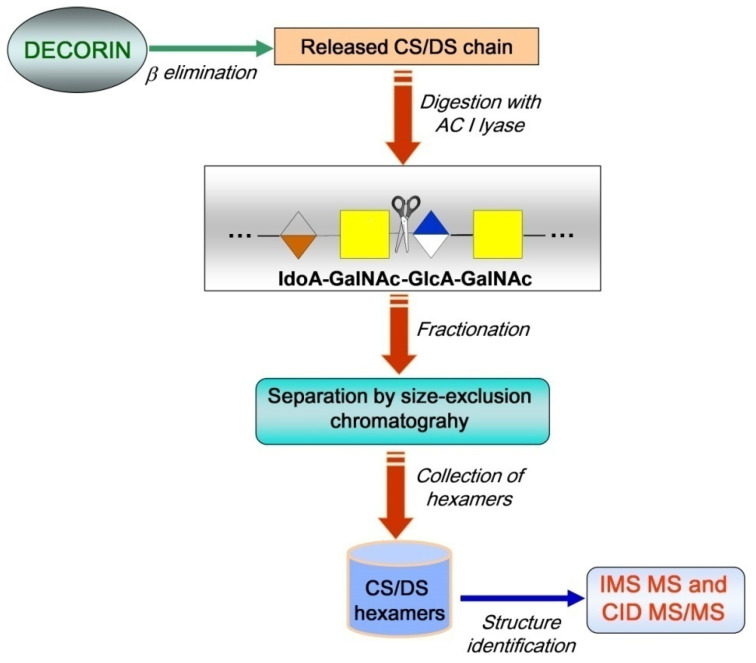
Schematic of the general workflow for CS/DS chain releasing, depolymerization by chondroitin AC I lyase, separation, collection, and analysis by IMS MS and CID MS/MS of hexasaccharide CS/DS domains in human DCN.

The IMS MS analysis of HEK293 DCN-derived CS/DS hexasaccharide domains followed the methodology presented in Figure 1. The strategy combines biochemical protocols and advanced analytical methods. The biochemical procedures were applied for CS/DS releasing from DCN by a β-elimination reaction and chain depolymerization using chondroitin AC I lyase, which specifically cleaves the glycosidic bond between GalNAc and D-glucuronate. The analytical step included separation by SEC, collection of hexasaccharides followed by compositional and structural analysis using IMS MS and CID MS/MS. The IMS MS and CID MS/MS targeted: 1. the separation of the analytes, based on ion mobilities, according to the charge state and the number of sulfates, leading to the discrimination of isobaric structures; 2. accurate measurement of the molecular masses of the components separated by IMS; and 3. determination of the sulfation code by fragmentation analysis.

**Figure 2:**
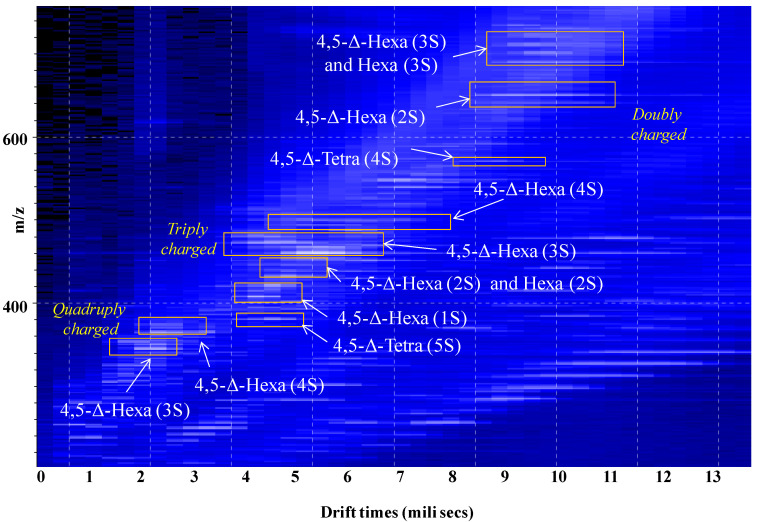
Driftscope display (drift time vs. *m/z*) of the total distribution of CS/DS hexasaccharide fraction ions. In the drift cell, the ions were separated based on the charge state and the number of sulfate groups. *n*S-number of SO_3_ groups.

A solution of 10 μL containing the hexasaccharides pooled after SEC was infused by (-) nanoESI into the Synapt G2S mass spectrometer and subjected to ion mobility separation followed by MS detection. The driftscope display, representing the drift time vs. *m/z* of the total distribution of CS/DS hexasaccharide ions separated by IMS, is presented in Figure 2. These results provide strong evidence that the various hexasaccharide species were not only separated according to the charge state, but also to the number of the sulfates in the molecule. Such a separation is of high importance for the accurate assignment of the ions since in ESI, because of CS/DS chain constitution, the monoisotopic signals corresponding to charge states that equal ½ the number of the disaccharide repeats are observed at the same *m/z* values. Hence, in the absence of the separation according to charge state and number of sulfates, these isobaric structures cannot be discriminated. For instance, if formed, the [M-3H]^3−^ of 4,5-Δ-GlcAGalNAc[IdoAGalNAc]_2_ (3S), the [M-2H]^2−^ of 4,5-Δ-GlcAGalNAc[IdoAGalNAc] (2S), and the [M-H]^−^ of 4,5-Δ-GlcAGalNAc (S), appear as signals at the same *m/z* 458.060 and only the separation based on ion mobility may discriminate the three isobaric structures. In our case, only the [M-3H]^3−^ of 4,5-Δ-GlcAGalNAc[IdoAGalNAc]_2_ (3S) was formed, and clearly evidenced through a single mobility feature revealing a triply charged species at *m/z* 458.064, which corresponds to the regularly sulfated unsaturated hexamer; however, as discussed below (Section 2.2.), IMS resolved isobaric species and avoided their simultaneous fragmentation by CID.

**Figure 3:**
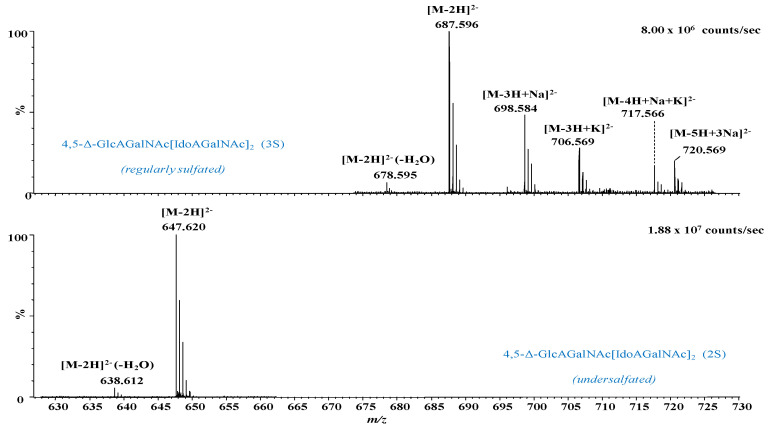
Extracted (-) nanoESI IMS mass spectra of doubly charged di- and trisulfated 4,5-Δ-GlcAGalNAc[IdoAGalNAc]_2_ from the corresponding areas indicated in Figure 2. *n*S-number of SO_3_ groups.

**Figure 4:**
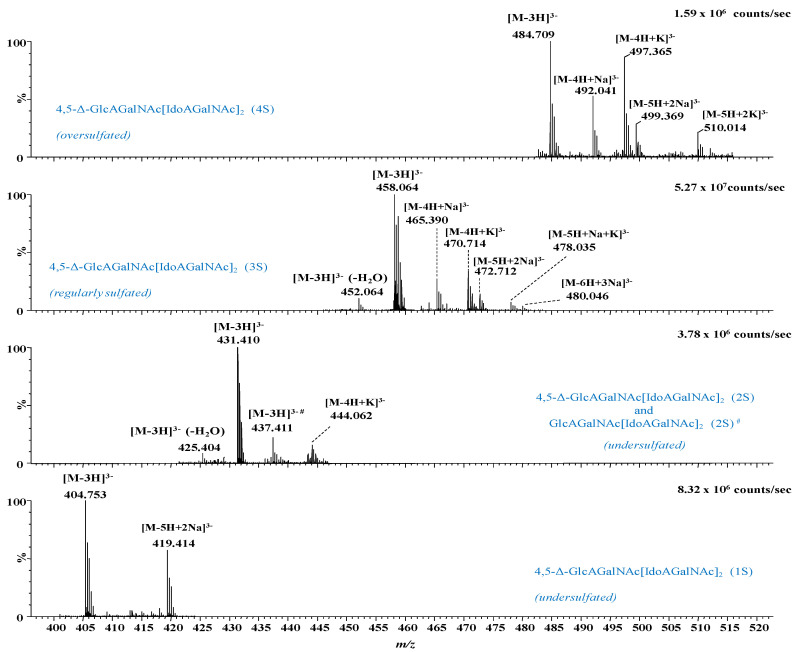
Extracted (-) nanoESI IMS mass spectra of triply charged mono- to tetrasulfated 4,5-Δ-GlcAGalNAc[IdoAGalNAc]_2_ from the corresponding areas indicated in Figure 2. *n*S-number of SO_3_ groups; #—saturated structure.

**Figure 5:**
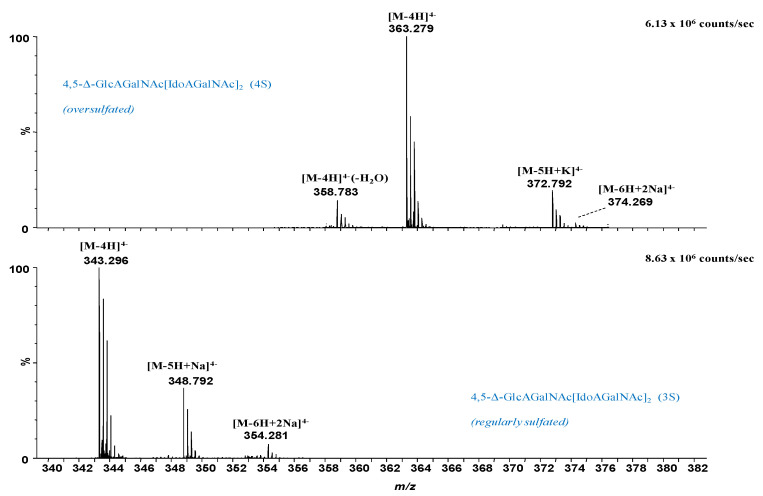
Extracted (-) nanoESI IMS mass spectra of quadruply charged tri- and tetrasulfated 4,5-Δ-GlcAGalNAc[IdoAGalNAc]_2_ from the corresponding areas indicated in Figure 2. *n*S-number of SO_3_ groups.

In Figure 3, Figure 4 and Figure 5 are presented, as example, the extracted mass spectra of the doubly charged di- and trisulfated 4,5-Δ-GlcAGalNAc[IdoAGalNAc]_2_, triply charged mono- to tetrasulfated 4,5-Δ-GlcAGalNAc[IdoAGalNAc]_2_ and quadruply charged tri- and tetrasulfated 4,5-Δ GlcAGalNAc[IdoAGalNAc]_2_ from the areas marked in the driftscope display, whereas in Table 1 are listed the 46 major ions detected in the hexasaccharide fraction, together with their assignment to unsaturated and saturated CS/DS species based on exact mass measurement and validation by MS/MS in some of the cases.

The spectral data analysis and interpretation have led to a number of findings that offer a new insight into the structural domains of HEK293 DCN-CS/DS. As visible in Figure 3, Figure 4 and Figure 5 and Table 1, the sample is dominated by hexasaccharide species of different sulfation patterns; since, to avoid the sample loss, the collected fraction was not rechromatographed, traces of tetrasaccharides were also detected.

Except for the common and most expected unsaturated hexasaccharide motif, which is the trisulfated 4,5-Δ-GlcAGalNAc[IdoAGalNAc]_2_, identified in no less than 18 anionic forms, with or without alkali adducts, and bearing from 1 to 4 negative charges, several unusual and biologically interesting under- and oversulfated as well as saturated structures were found.

From the technical point of view, the detection of oversulfated domains next to the overall elevated charging of the molecules and the formation of anionic species exhibiting all sulfate groups deprotonated (the number of charges equals the number of sulfates) indicate that the nanoESI conditions were properly optimized for a maximum reduction of the *in-source* loss of the labile sulfate ester group and of spectral artifacts, i.e., CS/DS species that have undergone a process of artificial *in-source* desulfation. Under these nanoESI IMS MS conditions, two oversulfated domains, both unsaturated, of which a hexasaccharide and a tetrasaccharide were identified in the collected fraction as follows: (i) the tetrasulfated 4,5-Δ-GlcAGalNAc[IdoAGalNAc]_2_ detected as quadruply, triply and doubly charged molecule in no less than 10 anionic forms, including the abundant [M-4H]^4−^ at *m/z* 363.279, and the ionic species with alkali adducts and dehydration by the loss of one H_2_O molecule (Table 1); and (ii) the pentasulfated unsaturated tetrasaccharide 4,5-Δ-GlcAGalNAcIdoAGalNAc identified as a [M-3H]^3−^ of high relative abundance at *m/z* 384.990.

Next to the oversulfated species, IMS MS also revealed two irregularly sulfated unsaturated hexasaccharide motifs characterized by undersulfation of the CS/DS chain; these are the monosulfated 4,5-Δ-GlcAGalNAc[IdoAGalNAc]_2_ and the bisulfated 4,5-Δ-GlcAGalNAc[IdoAGalNAc]_2_ detected as ions bearing from 1 to 4 negative charges. An important detail in the spectra and Table 1, is that the majority of these glycoforms, more exactly 45 out of 46 ions carry a double bond, demonstrating that the oligosaccharides originate from the non-reducing end of the CS/DS chain, being generated by the eliminative action of chondroitin AC I lyase on the GalNAc-GlcA linkages. On the other hand, one species, namely the undersulfated GlcAGalNAc[IdoAGalNAc]_2_ (2S) contains a saturated glucuronic acid at the non-reducing end, which is a “tag” straightforwardly recognizable by MS. A saturated HexA is specific for the terminus of the original CS/DS chain, since there is no chain extension by an additional GalNAc and no GalNAc-HexA linkage that would trigger the action of a lyase. Hence, the IMS MS data show that the non-reducing end of the entire GAG chain in HEK293 DCN is undersulfated, characterized by a structural motif consistent with a bisulfated hexasaccharide.

### 2.2. IMS CID MS/MS for the Determination of Misregulations in the Sulfation Code

Irregularities in the sulfation code of CS/DS domains, which are responsible for a variety of biological events, do not only mean over- and undersulfation, which are easily documented by accurate mass measurement following IMS MS separation and screening. The species considered regularly sulfated in that their mass corresponds to structures having the number of sulfates equal to the number of disaccharide repeats may, actually, present a misregulation of the sulfation pattern due to the unusual location of the sulfate groups in the chain.

In order to characterize possible misregulations in the sulfation code, which are rather due to the localization of the sulfates than their number, we have chosen for fragmentation analysis the [M-4H+K]^3−^ detected by IMS MS at *m/z* 470.714. By exact mass calculation, this ion was assigned to a trisulfated 4,5-Δ-GlcAGalNAc[IdoAGalNAc]_2_ species, considered regularly sulfated in terms of the number of SO_3_ groups. The option for this particular ion was guided by the fair relative intensity of the signal in the IMS MS and the previous observations [20,39,40] according to which, the loss of sulfates in CID is minimum for the precursor ions whose number of charges equals the number of the sulfate groups.

**Figure 6:**
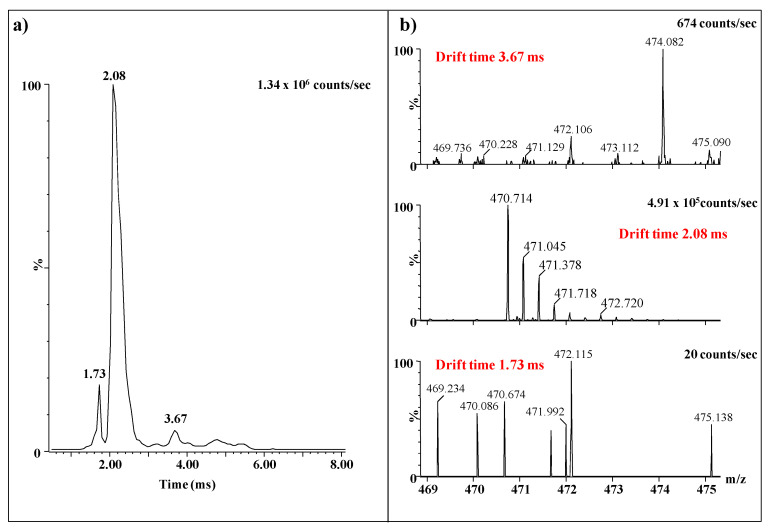
IMS MS of the precursor ion at *m/z* 470.714 fragmented by CID: (**a**) drift time distribution; (**b**) extracted mass spectra at the three mobility features: 3.67 ms, 2.08 ms, and 1.73 ms.

Except for the higher intensity of the signal, as compared to the MS screening without IMS, an interesting aspect related to this ion, which emphasizes once again the importance of IMS separation prior to MS and CID MS/MS is presented in Figure 6. Obviously, the drift time distribution for *m/z* 470.714 shows 3 mobility features at 3.67 ms, 2.08 ms, and 1.73 ms (Figure 6a). The extracted mass spectra (Figure 6b), for each of the signals, reveal that several different ions with values of the *m/z* close to 470.714 are present in the mixture and were separated based on their different mobility. However, only the ion with the drift time of 2.08 ms represents the triply charged precursor selected for fragmentation analysis. The other ions, of very low expression, are small molecules, most probably traces of impurities in the sample, detected due to their ionizability in the negative ion mode and the high sensitivity of the method. Without prior IMS separation, all ions would have been simultaneously sequenced generating a contradictory fragmentation spectrum impossible to interpret.

**Figure 7:**
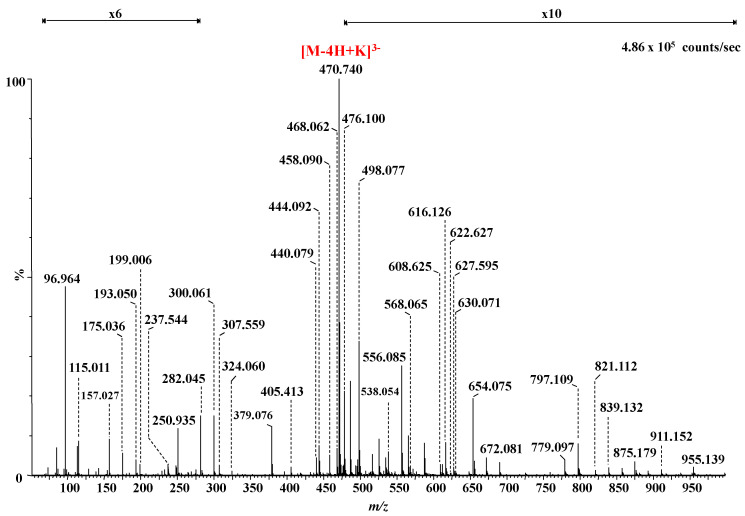
Negative ion mode nanoESI IMS CID MS/MS of the [M-4H+K]^3−^ detected at *m/z* 470.714 in the MS depicted in Figure 4, corresponding to trisulfated 4,5-Δ-GlcAGalNAc[IdoAGalNAc]_2_. Collision energy 30 eV. For a better visualization of the fragment ions, the areas *m/z* 100 to *m/z* 350 and *m/z* 475 to *m/z* 1000 were magnified ×6 and ×10, respectively.

The IMS CID MS/MS of the ion at *m/z* 470.714 is presented in Figure 7 whereas the fragment ions together with their assignment are listed in Table 2.

**Figure 8:**
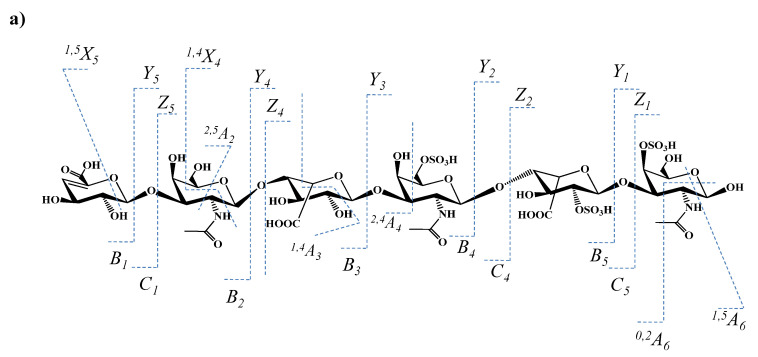

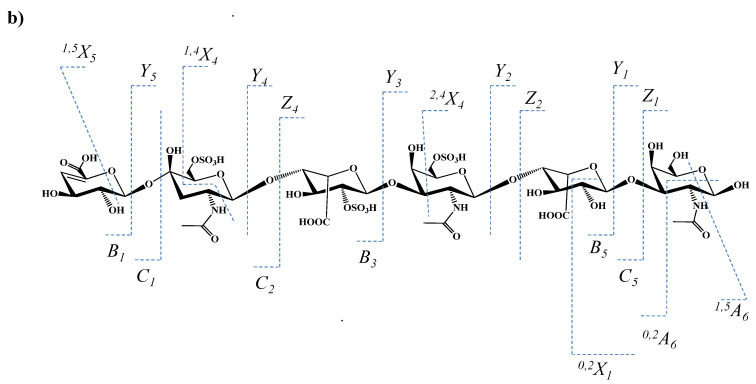
The two proposed structures with three sulfates located at the GalNAc-IdoA-GalNAc trisaccharide sequence from (**a**) the reducing or the (**b**) non-reducing end, together with the fragmentation pathways and the observed fragment ions for the trisulfated 4,5-Δ-GlcAGalNAc[IdoAGalNAc]_2_.

The MS/MS analysis targeted the evaluation of the sulfation code, in particular the determination of the sulfation sites along the chain. From this perspective, the generated fragment ions indicate in the first place that the non-reducing end of the molecule is unsulfated. The signals at *m/z* 405.413, 418.068, 432.071, 556.085, 612.035, 630.071, 654.075 correspond to no less than 7 ions generated by glycosidic bond or cross-ring cleavages supporting a GalNAc-IdoA-GalNAc-IdoA-GalNAc (3S) structure; this aspect illustrates that all three sulfates are positioned in the IdoA-rich pentasaccharide sequence and none at 4,5-Δ-GlcA. This concept is also supported by the oversulfated fragment ions at *m/z* 498.077, 507.085, and 997.154, which, according to mass calculation, have the composition IdoA-GalNAc-IdoA-GalNAc (3S). This sequence, which resulted by stepwise CID detachment of 4,5-Δ-GlcA and GalNAc, displays a higher number of SO_3_ groups than the number of GalNAc moieties in its constitution, being the first one to reveal that the investigated trisulfated hexasaccharide presents at least one structural irregularity due to the unusual position of a sulfate group. A further detailed analysis discloses two fragment ions at *m/z* 821.112 and 839.132 that are consistent with a motif having all three sulfates located at the GalNAc-IdoA-GalNAc trisaccharide sequence from either the reducing (Figure 8a) or the non-reducing end (Figure 8b). These findings prove that the structure of trisulfated 4,5-Δ-GlcAGalNAc[IdoAGalNAc]_2_, which, based on the MS screening, appeared initially as an ordinary motif within the CS/DS chain, exhibits in fact severe misregulations in the sulfation pattern.

The series of fragment ions detected by CID support both structures depicted in Figure 8. Due to the symmetry of the inner GalNAc-IdoA-GalNAc-IdoA-GalNAc motif and the lack of an aglycone to be used as a “tag”, no fragment ion able to support or completely rule out the structure in Figure 8a may be generated. On the other hand, on the basis of the previous observations related to the unsulfated 4,5-Δ-GlcA, the structure in Figure 8b is to be supported solely by fragment ions from the non-reducing end, having the nonsulfated 4,5-Δ-GlcA as a “tag”, to yield 4,5-Δ-GlcA-GalNAc-IdoA-GalNAc with all three sulfates located in GalNAc-IdoA-GalNAc.

In spite of the high sequence coverage provided by the CID experiment and the elevated mass accuracy of the generated fragment ions, none of the diagnostic ions was detected in the MS/MS of the trisulfated 4,5-Δ-GlcAGalNAc[IdoAGalNAc]_2_ species. However, given that the absence of a signal does not represent relevant information, in Figure 8a,b we propose two structural variants for the analyzed trisulfated hexasaccharide. However, since the IMS profile has shown a single mobility feature for the triply charged molecule at *m/z* 470.714, only one of these two glycoforms represents a genuine domain in the CS/DS chain. Without IMS, the occurrence of both isomeric motifs should have been considered. In view of the MS/MS issues presented above, we deem that the configuration presented in Figure 8a is the most probable one.

Additional structural information was provided by a number of detected ring-cleavage ions: (a) the doubly deprotonated ion at *m/z* 543.091, assigned to ^1,4^X_4_, documents that in the first GalNAc residue from the non-reducing end, C4 position is not sulfated in any of the two glycoforms shown in Figure 8; and (b) the monodeprotonated ^2,4^X_2_ for the structure in Figure 8a, or ^2,4^X_4_/Y_2_ for the structure in Figure 8b, detected at *m/z* 779.097, confirm that in any of the cases, the first GalNAc moiety from the non-reducing end is sulfated in position C6.

## 3. Materials and Methods

### 3.1. CS/DS Hexasaccharide Fraction

HEK293 cells were transfected with human decorin cDNA following the previously described protocol [28]. 500 μg of DCN were prepared as we have shown before [20]. Briefly, one liter of conditioned medium was collected; the secreted macromolecules were mixed with proteinase inhibitors, isolated on DEAE-Tris-Acryl M and concentrated with Aquacide I (Calbiochem, Bad Soden, Germany). PGs were dialyzed against 20mM Tris/HCl pH 7.4, containing 150mM NaCl and separated on a SEC-DEAE column. DCN purity was checked by silver stained 12.5% SDS-PAGE.

The CS/DS chain was released by β-elimination reaction in 200 μL of 0.15 M NaOH and 1 M NaBH_4_. The mixture was neutralized with acetic acid, diluted with 150 mM NaCl, 20 mM Tris-HCl, pH 7.4, and applied to a 0.5 mL DEAE-Tris-Acryl M (BioSepra, Cergy-Saint-Christophe, France) column prepared in a Pasteur pipet.

After washing with 150 mM NaCl, 20 mM Tris-HCl, pH 7.4, the GAG chains were eluted with 1.0 M NaCl, 20 mM Tris-HCl, pH 7.4, dialyzed against water, lyophilized and depolymerized by partial digestion with with 2 × 50 mU/assay chondroitin AC I lyase (Seikagaku Kogyo, Tokyo, Japan).Chondroitin AC I lyase specifically cleaves the linkage between GalNAc and D-GlcA irrespective of the sulfation code of the chain [20], via the neutralization of the negative charge of the carboxylic group, abstraction of the C-5 proton, and the elimination of the C4 hydroxyl group by introducing a C4–C5 double bond. Due to the specificity of AC I lyase, the origin of the Δ-HexA in the investigated sequences is known, even if, following the elimination reaction, the sterochemistry is lost. For this reason, since the origin of the HexA is known, for a better understanding, the Δ-HexA derived from GlcA is further denoted Δ-GlcA in the manuscript text.

The fractionation of the oligosaccharides was performed by SEC, on a Superdex Peptide HR10/30 column (Amersham-Pharmacia, Freiburg, Germany) as described by us before [20]. The hexasaccharide fraction was pooled and desalted by overnight dialysis using a prepacked D-Salt column (MWCO 5000) (Pierce, Rockford, IL, USA). To enhance the sample amount collected in the hexa fraction, the pool was not rechromatographed; therefore, as expected, the mixture contained traces of heterotetramers.

For the MS analysis the heterohexamer pool was dried to complete desiccation in a SpeedVac concentrator (SPD 111V-230, Thermo Electron, Asheville, NC, USA) coupled to a vacuum pump (PC 2002 Vario with CVC 2000 controller, Vaccubrand, Wertheim, Germany) and dissolved in pure methanol (Merck, Darmstadt, Germany) to a concentration of 10 pmol/μL, calculated for an average molecular weight of 1300 g/mol. Prior to the IMS MS analysis, the sample/methanol solution was briefly centrifuged in a Sigma 2–16 model centrifuge from Sartorius (Göttingen, Germany).

### 3.2. IMS MS and CID Tandem MS

The ion mobility mass spectrometry was performed on a Synapt G2S mass spectrometer (Waters, Manchester, UK) equipped with nanoESI source, tuned in the negative ion mode, and interfaced to a PC computer running the Waters MassLynx version V4.1, SCN 855, (Waters, Milford, MA, USA) and Waters Driftscope version V2.7software (Waters, Milford, MA, USA).

Ten µL solution of the hexasaccharide fraction, dissolved in pure methanol, were introduced into the back of a 10 cm long pulled emitter (ID 1.2 mm, OD 1.5 mm, 6.5 µm tip size, taper length 4 mm). For electrical contact, a 0.25 mm platinum wire was inserted into the solution. A steady spray and an efficient ionization of the CS/DS molecules, preventing the *in-source* loss of the labile SO_3_ group, were achieved by setting 1.4 kV on the nanoESI capillary and 15 V for the cone, respectively. All mass spectra were acquired in negative ion mode using a scan range from *m/z* 100 (to detected possible *in-source* fragmentation of 4,5-Δ-IdoA building block at *m/z* 157.027) to 2,500 and a scan time of 1.000 s at a resolution of 10,000 (for *m/z* 400).

For an effective separation, the MS and IMS parameters were set as follows: IMS gas flow 90 mL/min, IMS wave velocity 650 m/s, IMS wave height 40 V, source block temperature 120 °C, desolvation gas flow rate 500 L/h, and desolvation temperature 350 °C.

MS/MS experiments were carried out by CID using LM and HM parameters set to 4.7 and 15, respectively, and a collision energy of 30 eV. This value of the collision energy prevented an extensive desulfation and favored the generation of diagnostic fragment ions relevant for the sulfation code of the investigated molecule. The assignment of the fragment ions, generated by IMS CID MS/MS, followed the nomenclature introduced by Domon and Costello [41] and revised by Costello et al. [42]. In addition, the assignment to structures containing unsaturated GlcA was based on the specificity of chondroitin AC I lyase to form a 4,5 double bond, with the elimination of a water molecule in the cleaved GlcA moiety, i.e., the D-glucuronate at the non-reducing end. The resulting molecule is a hybrid CS/DS chain consisting of an unsaturated CS disaccharide having the structure 4,5-Δ-GlcA-GalNAc at the non-reducing end linked to a IdoA-rich motif (DS).

The five number of replicates, carried out for the experiment, have led to the following observations: (i) in terms of sensitivity, number of detected molecular/fragment ions, relative intensity, and charge state, the monitored in-run reproducibility of the experimental data, under identical set of conditions, was almost 100%; and (ii) the reproducibility from an experiment to another, performed using the same parameters, was 96%.

## 4. Conclusions

We have introduced in glycomics of hybrid CS/DS domains from human kidney cells DCN a robust methodology based on high-throughput IMS MS and MS/MS in combination with an efficient fragmentation technique based on collision-induced dissociation at low ion acceleration energies. The current research represents, basically, an extension of the previous work in the field, carried out by our group using capillary electrophoresis-MS and microfluidics-MS platforms developed for GAG profiling and functional interactomics involving CS/DS and growth factors.

The present protocol, connecting IMS separation, MS screening and tandem MS, allowed: (i) the discrimination of isobaric species by their separation according to the mobilities of their corresponding ions; (ii) the detection in the hexasaccharide domains of HEK293 cells DCN of novel over- and undersulfated CS/DS sequences which, in view of their sulfation status are, more likely, biologically active, and (iii) a detailed structural analysis including the identification of CS/DS sulfation code. Moreover, by IMS CID MS/MS, the misregulations in the SO_3_ distribution were also found. Fragmentation analysis data demonstrated that a trisulfated hexasaccharide domain, placed initially in the category of regular sequences due to the number of sulfates that equals the number of disaccharide repeats, corresponds, in fact, to an abnormally sulfated structure. The anomaly resides in the sulfate distribution at IdoA and the adjacent GalNAc residues, in a trisaccharide GalNAc-IdoA-GalNAc configuration in which every monosaccharide is monosulfated.

From the technical point of view, this approach, based on a versatile, fast, and integrated technique, offered a total analysis of hexa-CS/DS, at ultrahigh sensitivity, in a single run, in high-throughput mode. By performing an online separation, mass analysis, and fragmentation on a single instrument, IMS MS is able to cut across the laborious traditional methodologies existing in MS-based glycosaminoglycomics, which assume physical coupling of separation instruments to MS, with the entire arsenal of shortcomings that accompany such procedures.

Considering these results, we believe that the concept of the present approach addresses to a better extent the needs of glycosaminoglycomics for faster, more reliable, and more sensitive methodologies on one side and a few of the many questions still open, related to the structure and sulfation status of potential biologically active CS/DS domains in human decorin. On the other hand, it is noteworthy to mention that, in combination with ESI, neither the IMS method is able to eliminate the need to depolymerize the CS/DS chains and fractionate the samples prior to their structural analysis. The intermediate stages of digestion and fractionation, which follow the β-elimination, reduce the overall sensitivity of the methodology and only refer to analysis sequences of the CS/DS chain. For this reason, we consider that the perspectives of this field are related to the optimization and introduction in CS/DS research of matrix-assisted laser desorption/ionization (MALDI) IMS MS, which would allow a direct analysis of the entire chain.

## Figures and Tables

**Table 1:** Assignment of the major ions of CS/DS hexasaccharide fraction detected by IMS MS. *n*S-number of SO_3_ groups.

No.	*m/z* exp	*m/z* Theor	Mass Accuracy (ppm)	Proposed Stucture	Sulfation	Molecular Ion
1	305.040	305.037	−8.7	4,5-Δ-GlcAGalNAcIdoAGalNAc (2S)	2S	[M-3H]^3−^
2	314.309	314.306	−11.1	4,5-Δ-GlcAGalNAc[IdoAGalNAc]_2_ (1S)	1S	[M-6H+2Na]^4−^
3	323.308	323.304	−13.2	4,5-Δ-GlcAGalNAc[IdoAGalNAc]_2_ (2S)	2S	[M-4H]^4−^
4	324.301	324.297	−13.9	4,5-Δ-GlcAGalNAc[IdoAGalNAc]_2_ - H_2_O (2S)	2S	[M-5H+Na]^4−^
5	343.296	343.293	−8.7	4,5-Δ-GlcAGalNAc[IdoAGalNAc]_2_ (3S)	3S	[M-4H]^4−^
6	348.792	348.789	−10.1	4,5-Δ-GlcAGalNAc[IdoAGalNAc]_2_ (3S)	3S	[M-5H+Na]^4−^
7	352.785	352.782	−9.2	4,5-Δ-GlcAGalNAc[IdoAGalNAc]_2_ (3S)	3S	[M-3H+K]^2−^
8	354.281	354.284	8.5	4,5-Δ-GlcAGalNAc[IdoAGalNAc]_2_ (3S)	3S	[M-6H+2Na]^4−^
9	358.783	358.780	−9.8	4,5-Δ-GlcAGalNAc[IdoAGalNAc]_2_ - H_2_O (4S)	4S	[M-4H]^4−^
10	363.279	363.282	9.0	4,5-Δ-GlcAGalNAc[IdoAGalNAc]_2_ (4S)	4S	[M-4H]^4−^
11	372.774	372.771	−8.1	4,5-Δ-GlcAGalNAc[IdoAGalNAc]_2_ (4S)	4S	[M-5H+K]^4−^
12	374.269	374.273	11.4	4,5-Δ-GlcAGalNAc[IdoAGalNAc]_2_ (4S)	4S	[M-6H+2Na]^4−^
13	384.990	384.994	11.3	4,5-Δ-GlcAGalNAcIdoAGalNAc (5S)	5S	[M-3H]^3−^
14	404.753	404.755	5.8	4,5-Δ-GlcAGalNAc[IdoAGalNAc]_2_ (1S)	1S	[M-3H]^3−^
15	419.414	419.410	−9.5	4,5-Δ-GlcAGalNAc[IdoAGalNAc]_2_ (1S)	1S	[M-5H+2Na]^3−^
16	425.404	425.408	8.6	4,5-Δ-GlcAGalNAc[IdoAGalNAc]_2_ - H_2_O (2S)	2S	[M-3H]^3−^
17	431.410	431.408	−5.4	4,5-Δ-GlcAGalNAc[IdoAGalNAc]_2_ (2S)	2S	[M-3H]^3−^
18	437.411	437.408	−7.6	GlcAGalNAc[IdoAGalNAc]_2_ (2S)	2S	[M-3H]^3−^
19	444.062	444.059	−6.0	4,5-Δ-GlcAGalNAc[IdoAGalNAc]_2_ (2S)	2S	[M-4H+K]^3−^
20	449.064	449.060	−8.9	4,5-Δ-GlcAGalNAcIdoAGalNAc - H_2_O (2S)	2S	[M-2H]^2−^
21	452.064	452.060	−8.8	4,5-Δ-GlcAGalNAc[IdoAGalNAc]_2_ - H_2_O (3S)	3S	[M-3H]^3−^
22	458.064	458.060	−8.7	4,5-Δ-GlcAGalNAc[IdoAGalNAc]_2_ (3S)	3S	[M-3H]^3−^
23	465.390	465.387	−5.7	4,5-Δ-GlcAGalNAc[IdoAGalNAc]_2_ (3S)	3S	[M-4H+Na]^3−^
24	470.714	470.712	−5.0	4,5-Δ-GlcAGalNAc[IdoAGalNAc]_2_ (3S)	3S	[M-4H+K]^3−^
25	472.712	472.715	5.6	4,5-Δ-GlcAGalNAc[IdoAGalNAc]_2_ (3S)	3S	[M-5H+2Na]^3−^
26	478.035	478.039	8.4	4,5-Δ-GlcAGalNAc[IdoAGalNAc]_2_ (3S)	3S	[M-5H+Na+K]^3−^
27	480.046	480.042	−8.3	4,5-Δ-GlcAGalNAc[IdoAGalNAc]_2_ (3S)	3S	[M-6H+3Na]^3−^
28	484.709	484.712	6.9	4,5-Δ-GlcAGalNAc[IdoAGalNAc]_2_ (4S)	4S	[M-3H]^3−^
29	492.041	492.040	−2.7	4,5-Δ-GlcAGalNAc[IdoAGalNAc]_2_ (4S)	4S	[M-4H+Na]^3−^
30	497.365	497.364	−2.0	4,5-Δ-GlcAGalNAc[IdoAGalNAc]_2_ (4S)	4S	[M-4H+K]^3−^
31	499.369	499.367	−4.0	4,5-Δ-GlcAGalNAc[IdoAGalNAc]_2_ (4S)	4S	[M-5H+2Na]^3−^
32	510.014	510.016	3.9	4,5-Δ-GlcAGalNAc[IdoAGalNAc]_2_ (4S)	4S	[M-5H+2K]^3−^
33	570.994	570.990	−7.0	4,5-Δ-GlcAGalNAcIdoAGalNAc (4S)	4S	[M-5H+3Na]^2−^
34	629.617	629.619	3.2	4,5-Δ-GlcAGalNAc[IdoAGalNAc]_2_ (1S)	1S	[M-4H+2Na]^2−^
35	638.612	638.610	−3.1	4,5-Δ-GlcAGalNAc[IdoAGalNAc]_2_ - H_2_O (2S)	2S	[M-2H]^2−^
36	647.620	647.616	−7.0	4,5-Δ-GlcAGalNAc[IdoAGalNAc]_2_ (2S)	2S	[M-2H]^2−^
37	656.626	656.621	−7.6	GlcAGalNAc[IdoAGalNAc]_2_ (2S)	2S	[M-2H]^2−^
38	678.595	678.589	−8.8	4,5-Δ-GlcAGalNAc[IdoAGalNAc]_2_ - H_2_O (3S)	3S	[M-2H]^2−^
39	687.596	687.594	−2.9	4,5-Δ-GlcAGalNAc[IdoAGalNAc]_2_ (3S)	3S	[M-2H]^2−^
40	698.584	698.585	1.4	4,5-Δ-GlcAGalNAc[IdoAGalNAc]_2_ (3S)	3S	[M-3H+Na]^2−^
41	706.569	706.572	4.2	4,5-Δ-GlcAGalNAc[IdoAGalNAc]_2_ (3S)	3S	[M-3H+K]^2−^
42	717.566	717.563	−4.9	4,5-Δ-GlcAGalNAc[IdoAGalNAc]_2_ (3S)	3S	[M-4H+Na+K]^2−^
43	720.569	720.567	−2.8	4,5-Δ-GlcAGalNAc[IdoAGalNAc]_2_ (3S)	3S	[M-5H+3Na]^2−^
44	899.133	899.128	−5.6	4,5-Δ-GlcAGalNAcIdoAGalNAc - 2H_2_O (2S)	2S	[M-2H]^2−^
45	1296.242	1296.239	−2.3	4,5-Δ-GlcAGalNAc[IdoAGalNAc]_2_ (2S)	2S	[M-H]^−^
46	1376.198	1376.196	−1.5	4,5-Δ-GlcAGalNAc[IdoAGalNAc]_2_ (3S)	3S	[M-H]^−^

**Table 2:** *m/z* values of the fragment ions generated by CID MS/MS experiment in Figure 7 on the trisulfated hexasaccharide detected at *m/z* 470.714 in the IMS MS and their structure assignment. *n*S denotes the number of sulfate groups in fragment ions. ^a^ Regular sequence ions; ^b^ Oversulfated fragment ions.

*m/z*	Charge State	Structure	The Type of Ion for the Structure in	
			Figure 8a	Figure 8b
96.964	1	HOSO_3_		
115.011	1	GalNAc	^2,5^X_0_	
157.027	1	4,5-Δ-GlcA	B_1_	
175.036	1	4,5-Δ-GlcA	C_1_	
193.050	1	IdoA (0S)	Y_2_/Z_1 or_ Y_4_/Z_3_	
199.006	1	GalNAc (1S)	^0,2^A_6_/B_5_ or^0,2^A_4_/B_3_	
237.544	1	IdoA (1S) ^b^	Z_2_/Y_1_	Z_4_/Y_3_
282.045	1	GalNAc (1S) ^a^	Z_1_	Z_5_/Z_4_
300.061	1	GalNAc (1S) ^a^	Y_1_	Y_5_/Z_4_
307.559	2	IdoAGalNAcIdoA (1S) ^a^	Z_4_/Y_1_	B_3_
343.318	1	IdoAGalNAc (1S) ^a^	^0,2^X_1_	^2,5^A_2_
379.076	2	GalNAcIdoAGalNAc (2S) ^a^	Y_3_	Z_5_/Z_2_
405.413	3	GalNAcIdoAGalNAcIdoAGalNAc (3S) ^a^	Y_5_	
418.068	3	GalNAcIdoAGalNAcIdoAGalNAc (3S) ^a^	Y_5_+K	
432.071	3	GalNAcIdoAGalNAcIdoAGalNAc (3S) ^a^	^2,5^A_6_ +K	
440.079	1	4,5-Δ-GlcAGalNAc (1S) or IdoAGalNAc (1S) ^a^	Z_4_/Y_2_	B_2_
444.092	3	GlcAGalNAcIdoAGalNAcIdoAGalNAc (3S) ^a^	^1,5^A_6_	^1,5^A_6_
454.023	1	IdoAGalNAc (2S) ^b^	^0,3^X_1_	^0,3^X_3_/Z_2_
458.090	3	GlcAGalNAcIdoAGalNAcIdoAGalNAc (3S) ^a^	[M-3H]^3−^	
468.062	2	IdoAGalNAcIdoAGalNAc (2S) ^a^	Y_4_	
470.714	3	4,5-Δ-GlcAGalNAcIdoAGalNAcIdoAGalNAc (3S) ^a^	[M-4H+K]^3−^	
476.100	1	IdoAGalNAc (1S) ^a^	Y_4_/Z_2_	Y_3/_Z_1_ orY_4_/Z_2_
498.077	2	IdoAGalNAcIdoAGalNAc (3S) ^b^	Z_4_	C_4_
507.085	2	IdoAGalNAcIdoAGalNAc (3S) ^b^	Y_4_	Y_5/_Z_1_
538.054	1	4,5-Δ-GlcAGalNAcIdoA (0S)	B_3_	
		IdoAGalNAc (2S) ^b^	Z_2_	Y_4/_Y_2_
543.091	2	GalNAcIdoAGalNAcIdoAGalNAc (3S) ^a^	^1,4^X_4_	
556.085	2	4,5-Δ-GlcAGalNAcIdoAGalNAcIdoA (2S) ^a^	B_5_ +K	
565.092	2	4,5-Δ-GlcAGalNAcIdoAGalNAcIdoA (2S) ^a^	C_5_ +K	
568.065	2	GalNAcIdoAGalNAcIdoAGalNAc (2S)	Y_5_	
608.625	2	GalNAcIdoAGalNAcIdoAGalNAc (3S) ^a^	Y_5_	
612.035	1	IdoAGalNAcIdoA (2S) ^b^	^1,4^A_5/_C_2_	^1,4^A_3_
616.126	1	4,5-Δ-GlcAGalNAcIdoA (1S) ^a^ IdoAGalNAcIdoA (1S) ^a^	- Z_4_/Y_1_	B_3_ Z_4_/Y_1_
622.627	2	4,5-Δ-GlcAGalNAcIdoAGalNAcIdoAGalNAc (3S) ^a^	^1,5^X_5_	
627.595	2	GalNAcIdoAGalNAcIdoAGalNAc (3S) ^a^	Y_5_+K	
630.071	2	4,5-Δ-GlcAGalNAcIdoAGalNAcIdoAGalNAc (3S) ^a^	^2,5^A_6_	
648.613	2	GalNAcIdoAGalNAcIdoAGalNAc (3S) ^a^	^2,5^A_6_ +K	
654.075	1	IdoAGalNAcIdoA (1S) ^a^	Z_4_/Y_1_+K	B_3_+K
779.097	1	GalNAcIdoAGalNAc (3S) ^b^	^2,4^X_2_	^2,4^X_4_/Y_2_
797.109	1	GalNAcIdoAGalNAc (2S) ^a^	Y_3_+K	Y_5_/Z_2_+K
821.112	1	GalNAcIdoAGalNAc (3S) ^b^	Z_3_	Y_5_/Y_2_
839.132	1	GalNAcIdoAGalNAc (3S) ^b^	Y_3_	Y_5_/Z_2_
857.169	1	4,5-Δ-GlcAGalNAcIdoAGalNAc (1S)	B_4_+K	B_4_+K or Y_5_/Y_1_+K
875.179	1	4,5-Δ-GlcAGalNAcIdoAGalNAc (1S)	C_4_+K	C_4_+K or Y_5_/Z_1_+K
911.152	1	IdoAGalNAcIdoAGalNAc (3S) ^b^	^0,3^X_3_	C_4_/^0,3^A_1_
917.187	1	IdoAGalNAcIdoAGalNAc (2S) ^a^	Z_4_	Z_4_
955.139	1	IdoAGalNAcIdoAGalNAc (3S) ^b^ IdoAGalNAcIdoAGalNAc (2S) ^a^	^2,4^X_3_ Z_4_+K	^2,4^X_4_/Z_1_ Z_4_+K
997.154	1	IdoAGalNAcIdoAGalNAc (3S) ^b^	Z_4_	Y_5_/Y_1_

## Data Availability

Not applicable.
